# Pediatric Lichen: Epidemiological and Clinical Characteristics in Children

**DOI:** 10.7759/cureus.108386

**Published:** 2026-05-06

**Authors:** Imane Hakim, Layla Bendaoud, Maryem Aboudourib, Said Amal, Ouafa Hocar

**Affiliations:** 1 Department of Dermatology, Mohammed VI University Hospital, Biosciences Laboratory, Faculty of Medicine and Pharmacy, Marrakech, MAR

**Keywords:** clinicodermoscopic correlation, dermoscopy, inflammatory dermatoses, lichen pigmentosus, lichen planus, lichen sclerosus, pediatric dermatology, pediatric lichen

## Abstract

Background: Lichen and related lichenoid dermatoses, including lichen planus and lichen sclerosus, are uncommon in childhood and remain insufficiently characterized due to their clinical heterogeneity and potential functional and psychological impact.

Objective: To describe the epidemiological, clinical, dermoscopic, and therapeutic characteristics of pediatric lichen in a Moroccan tertiary dermatology center.

Methods: We conducted a retrospective descriptive study in the pediatric dermatology outpatient clinic of the Dermatology Department of Mohammed VI University Hospital, Marrakech, over a period of 14 years and 8 months (January 2010 to August 2024). Diagnosis was based on clinical and histological criteria after exclusion of differential diagnoses. Dermoscopic evaluation was performed using standard dermoscopic examination.

Results: Thirty-six children were included. The mean age was 10 years (range: 4-16), with a marked female predominance (75%). Lichen sclerosus was the most frequent subtype. Dermoscopy revealed reproducible patterns according to clinical variants, contributing to diagnostic orientation. Associated conditions included alopecia areata, vitiligo, and linear morphea.

Conclusion: Pediatric lichen is a rare and heterogeneous condition in which non-classical forms may predominate. Clinicodermoscopic evaluation appears to be a valuable non-invasive tool to improve diagnostic accuracy and guide management in children.

## Introduction

Lichen is a chronic inflammatory dermatosis that may involve the skin, adnexa, mucous membranes, and, depending on the subtype, the scalp and genital area. In daily practice, it is classically regarded as a disease of adults, especially middle-aged patients. In childhood, however, it is uncommon and often underrecognized because of its clinical polymorphism, its overlap with other inflammatory or pigmentary dermatoses, and the variable awareness of pediatric presentations among clinicians [1,2]. This rarity explains why most available data come from isolated case reports, small retrospective series, or reviews compiling heterogeneous phenotypes rather than from large prospective pediatric cohorts.

Beyond its rarity, pediatric lichen is clinically important because some subtypes may lead to long-term sequelae. Genital forms may be associated with discomfort, pruritus, or anatomical changes in severe cases; nail involvement may result in permanent dystrophy; and scalp disease may progress toward cicatricial alopecia if not recognized early [3,4]. Pigmentary forms are often a source of aesthetic concern and may markedly affect quality of life in darker phototypes. These considerations justify a better characterization of pediatric disease and a pragmatic approach integrating clinicopathological correlation with non-invasive tools such as dermoscopy.

Dermoscopy has progressively become a useful adjunct in inflammatory dermatoses. In lichen, it provides clues that may orient diagnosis, support subtype classification, and help select the most representative lesion for biopsy when histologic confirmation is needed. In a pediatric setting, this non-invasive contribution is particularly valuable because it may limit unnecessary procedures and facilitate follow-up [5].

The objective of the present study was therefore to describe the epidemiological profile, clinical spectrum, dermoscopic findings, associated conditions, and therapeutic issues of pediatric lichen managed in a tertiary dermatology department, including a dedicated pediatric dermatology consultation, in Marrakech over nearly 15 years.

## Materials and methods

Study design and setting

We conducted a retrospective descriptive study in the pediatric dermatology consultation unit of the Department of Dermatology of Mohammed VI University Hospital, Marrakech, Morocco. The study was carried out over a period of 14 years and 8 months, from January 2010 to August 2024.

Study population

The study included pediatric patients aged ≤16 years who were diagnosed with lichen and related lichenoid dermatoses during the study period and managed in our department.

Inclusion criteria

Patients were included if they fulfilled the following criteria: age ≤16 years and diagnosis of lichen based on clinical examination, supported by histopathological confirmation when required. Only patients with sufficient clinical data allowing analysis of epidemiological, clinical, dermoscopic, and therapeutic features were retained.

Exclusion criteria

Patients were excluded in cases of incomplete medical records, uncertain diagnosis, lack of sufficient clinical data, or presence of other dermatoses mimicking lichen. These exclusions may represent a potential source of selection bias inherent to the retrospective design of the study. 

Data collection

Data were collected retrospectively from medical records. The following variables were analyzed: age at diagnosis, sex, phototype, duration of disease before consultation, clinical subtype, localization, dermoscopic findings, associated diseases, and therapeutic management.

Dermoscopic evaluation

Dermoscopic examination was performed as part of routine clinical practice using a DermLite 4 device (DermLite LLC, Aliso Viejo, CA). The main dermoscopic patterns were recorded according to the clinical subtype of lichen.

Statistical analysis

Data were analyzed using descriptive statistics. Quantitative variables were expressed as mean ± standard deviation (SD), while qualitative variables were expressed as absolute numbers and percentages (n (%)). 

Ethical considerations

This retrospective study was conducted in accordance with ethical standards. Patient anonymity was strictly preserved, and all data were handled confidentially.

## Results

A total of 36 children were included in the study. The mean age at diagnosis was 9.9 ± 3.2 years, with extremes ranging from 4 to 16 years. There was a marked female predominance, with girls representing 75% of the cohort and boys 25%, corresponding to a sex ratio of 0.33. Phototype IV was the most frequent, accounting for 58.82% of patients, followed by phototype III in 41.17%. The mean duration of evolution before diagnosis was 19.4 months. Epidemiological characteristics of the study population are summarized in Table 1.

**Table 1:** Epidemiological characteristics of the study population Data are presented as n (%).

Variable	Result
Number of cases	36
Mean age	9.9 ± 3.2 years (range: 4-16)
Female sex	27 (75%)
Male sex	9 (25%)
Sex ratio (M/F)	0.33
Phototype IV	21 (58.82%)
Phototype III	15 (41.17%)
Mean duration before diagnosis	19.4 months

**Figure 1:**
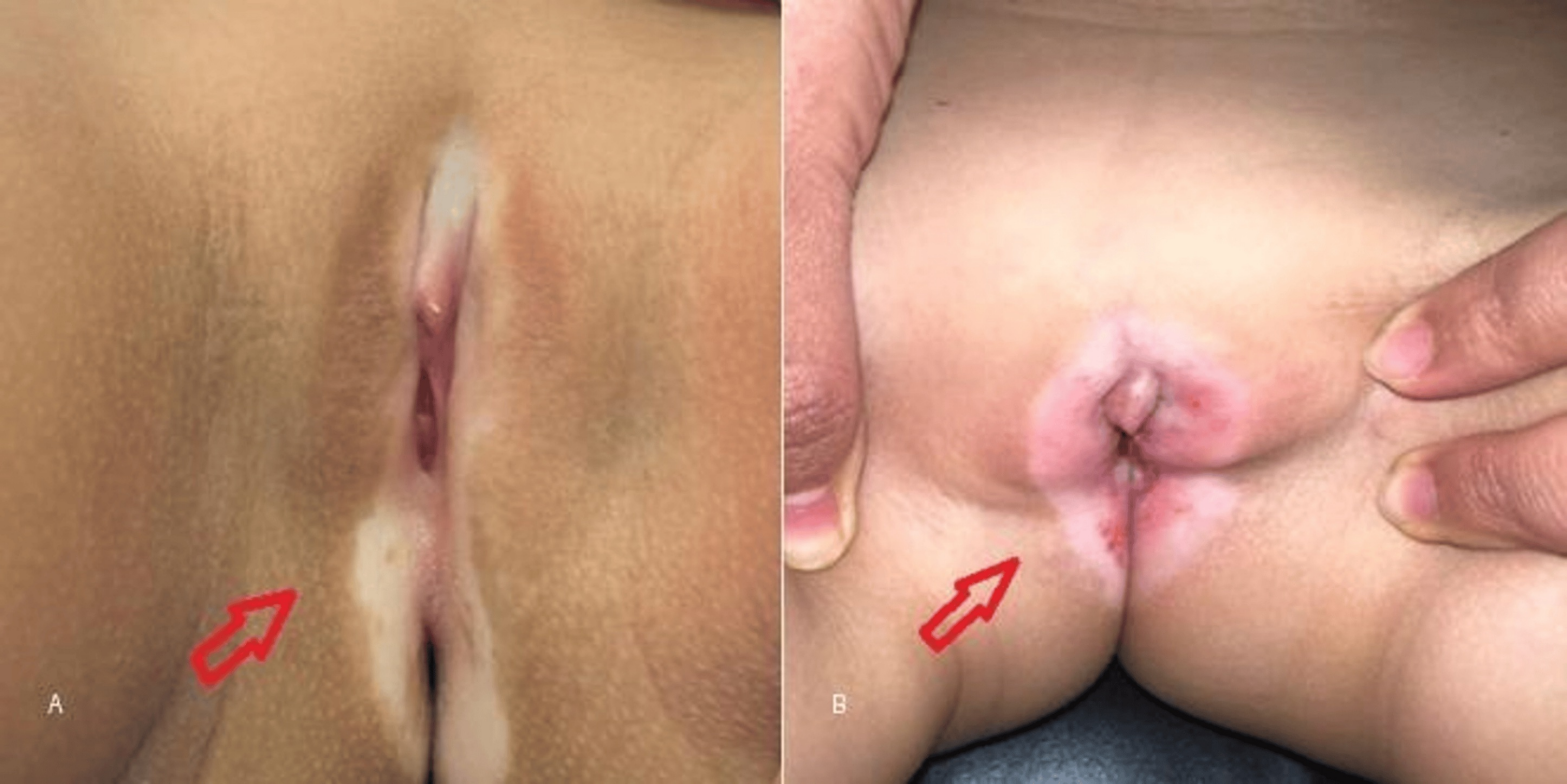
Clinical features of pediatric genital lichen sclerosus. (A) Hypopigmented atrophic plaques involving the vulvar region (arrow). (B) Erythema and fissuring reflecting active inflammatory involvement (arrow).

**Figure 2:**
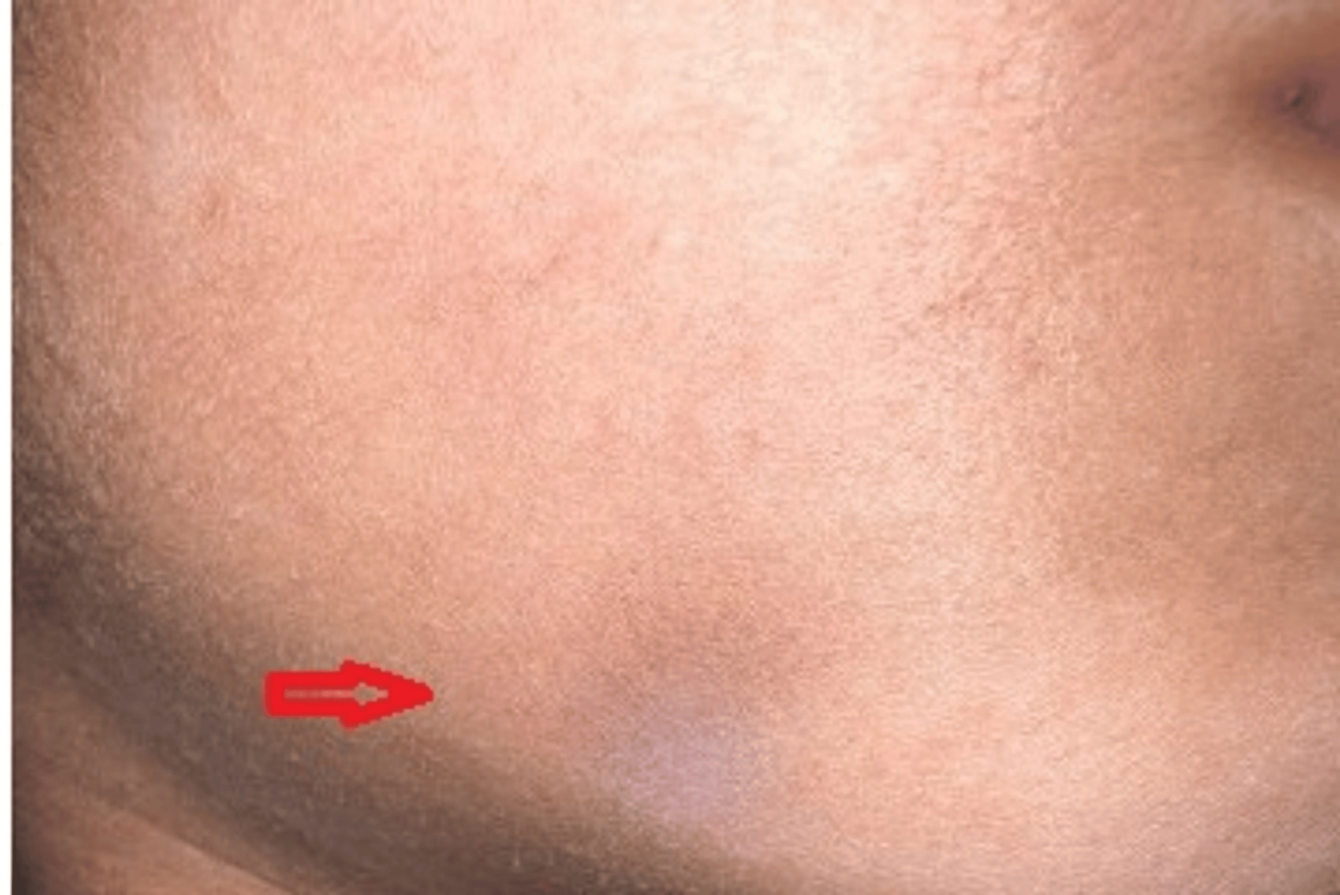
Representative clinical image of lichen pigmentosus on the mandibular region.

**Figure 3:**
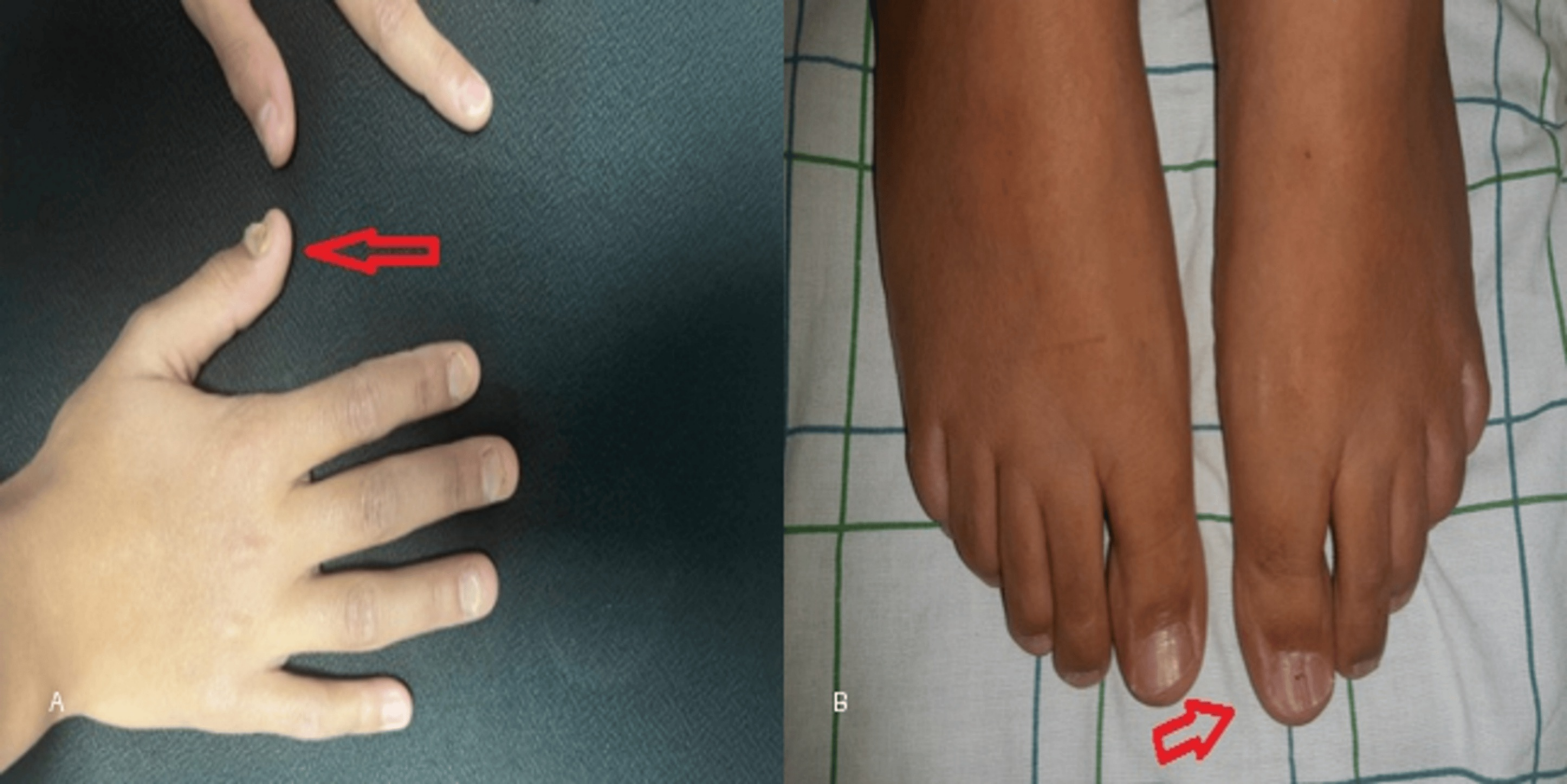
Clinical features of nail involvement in pediatric lichen planus. (A) Fingernail dystrophy with longitudinal ridging and nail plate alteration (arrow). (B) Toenail dystrophy reflecting similar involvement (arrow).

**Figure 4:**
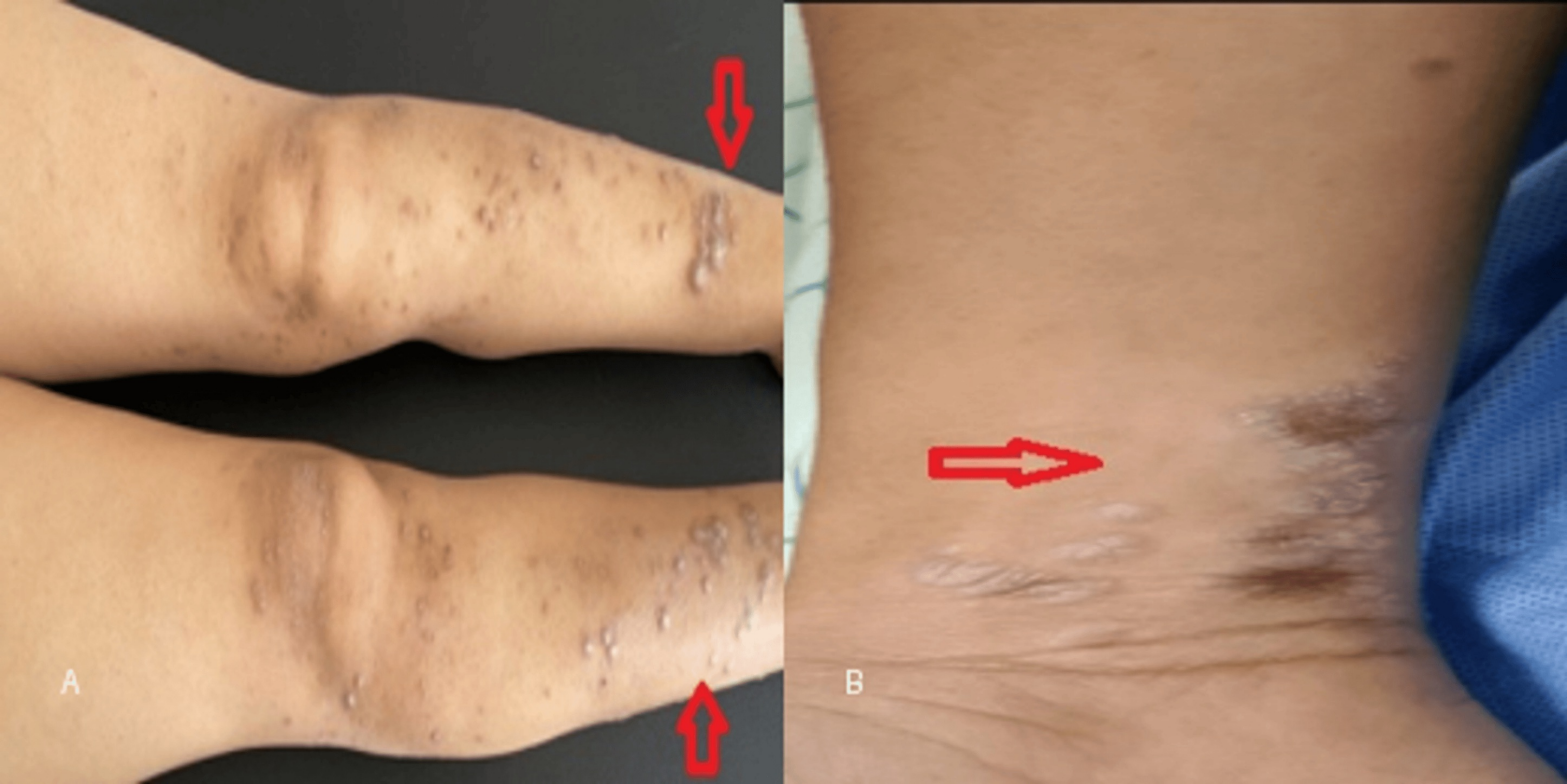
Representative clinical images of classic lichen planus. (A) Multiple violaceous papules with post-inflammatory hyperpigmentation on the lower limbs (arrow). (B) Well-defined violaceous plaque with a slightly scaly surface (arrow).

**Figure 5:**
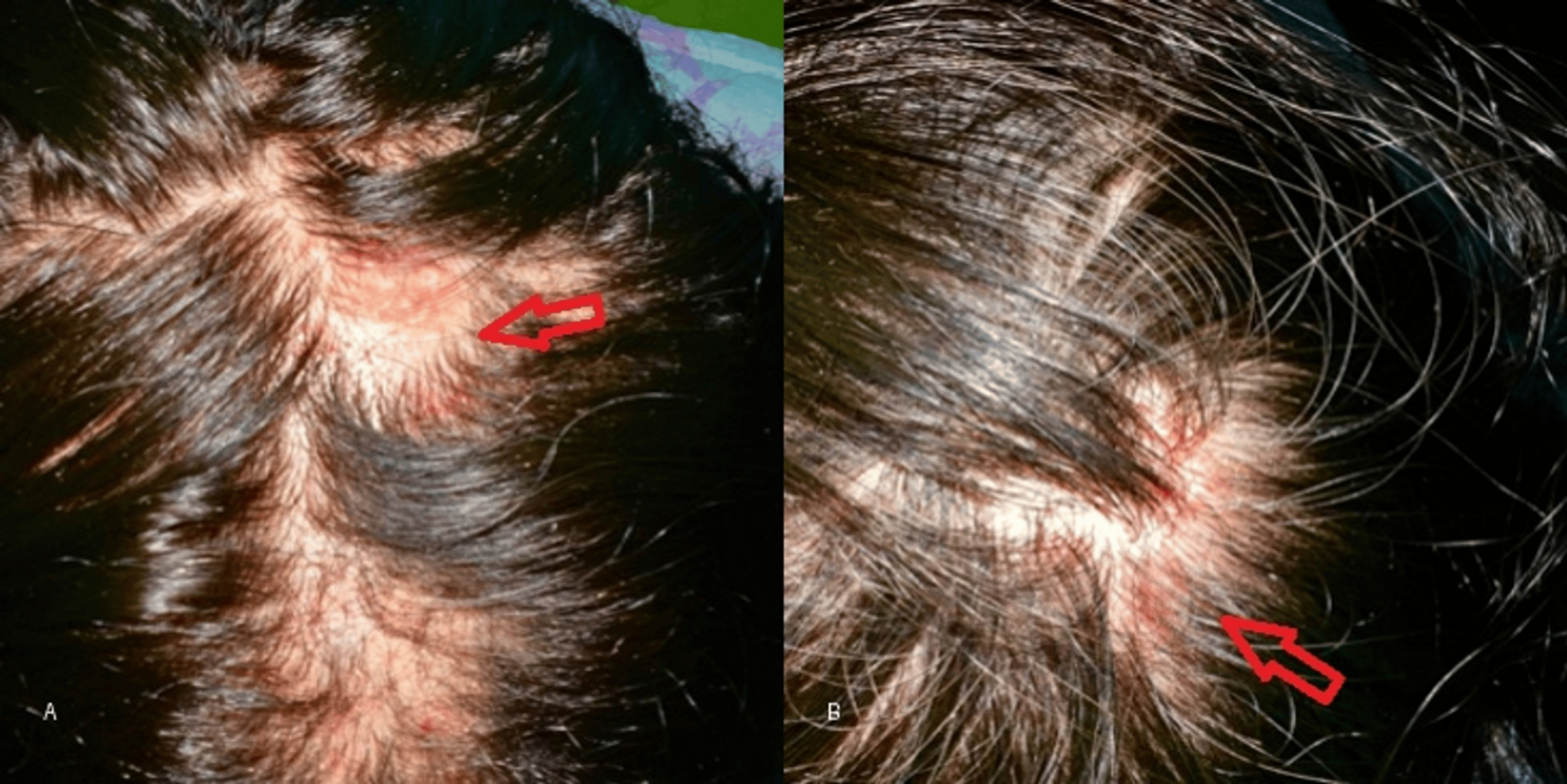
Representative clinical images of lichen planopilaris. (A) Erythematous scalp lesion with perifollicular inflammation (arrow). (B) Localized erythematous area with perifollicular scaling, consistent with active inflammation (arrow).

The distribution of clinical subtypes showed that childhood lichen was far from limited to classic lichen planus. Lichen sclerosus was the most frequent presentation, representing 36.11% of the cases (Figure 1). It was followed by lichen pigmentosus in 27.78% (Figure 2), nail lichen in 19.44% (Figure 3), classic lichen planus in 13.88% (Figure 4), and lichen planopilaris in 2.77% (Figure 5).

The distribution of clinical forms of pediatric lichen in the present series is summarized in Table 2.

**Table 2:** Clinical forms of pediatric lichen in the present series Data are presented as n (%).

Subtype	Frequency
Lichen sclerosus	13 (36.11%)
Lichen pigmentosus	10 (27.78%)
Nail lichen	7 (19.44%)
Classic lichen planus	5 (13.88%)
Lichen planopilaris	1 (2.77%)

Dermoscopy revealed features that differed according to subtype. In lichen pigmentosus, the main findings were a brown background, brown dots, brown globules, a reticular pattern, and an arciform pattern. In classic lichen planus, the principal findings were Wickham striae, radial linear vessels, and dotted vessels. In lichen planopilaris, dermoscopy showed white-pink areas, scaling, perifollicular erythema, and peripilar casts. In nail lichen, the described findings were onycholysis, subungual hyperkeratosis, and splinter hemorrhages. Dermoscopic findings according to lichen subtype are summarized in Table 3.

**Table 3:** Dermoscopic findings according to lichen subtype

Subtype	Dermoscopic findings
Lichen pigmentosus	Brown background, brown dots, brown globules, reticular pattern, arciform pattern
Classic lichen planus	Wickham striae, radial linear vessels, dotted vessels
Lichen planopilaris	White-pink areas, scaling, perifollicular erythema, peripilar casts
Nail lichen	Onycholysis, subungual hyperkeratosis, splinter hemorrhages

**Figure 6:**
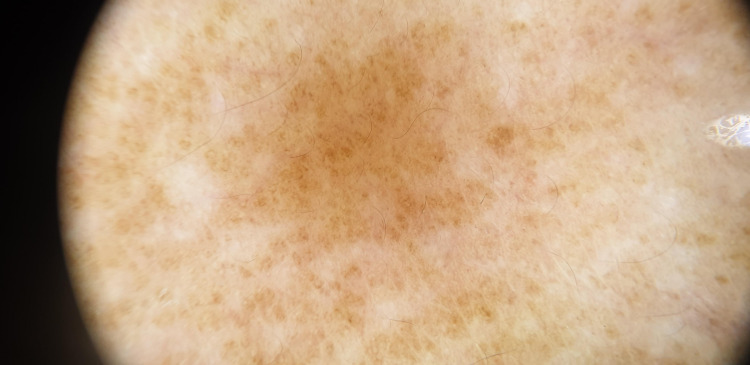
Dermoscopy of lichen pigmentosus demonstrating a diffuse brown background with multiple brown dots and globules, forming a granular and reticular pigmentary pattern.

**Figure 7:**
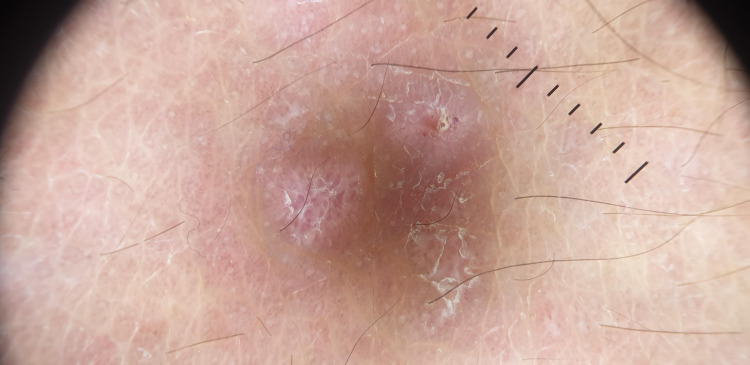
Dermoscopy of classic lichen planus demonstrating Wickham striae on an erythematous background associated with fine scaling and structureless whitish areas.

**Figure 8:**
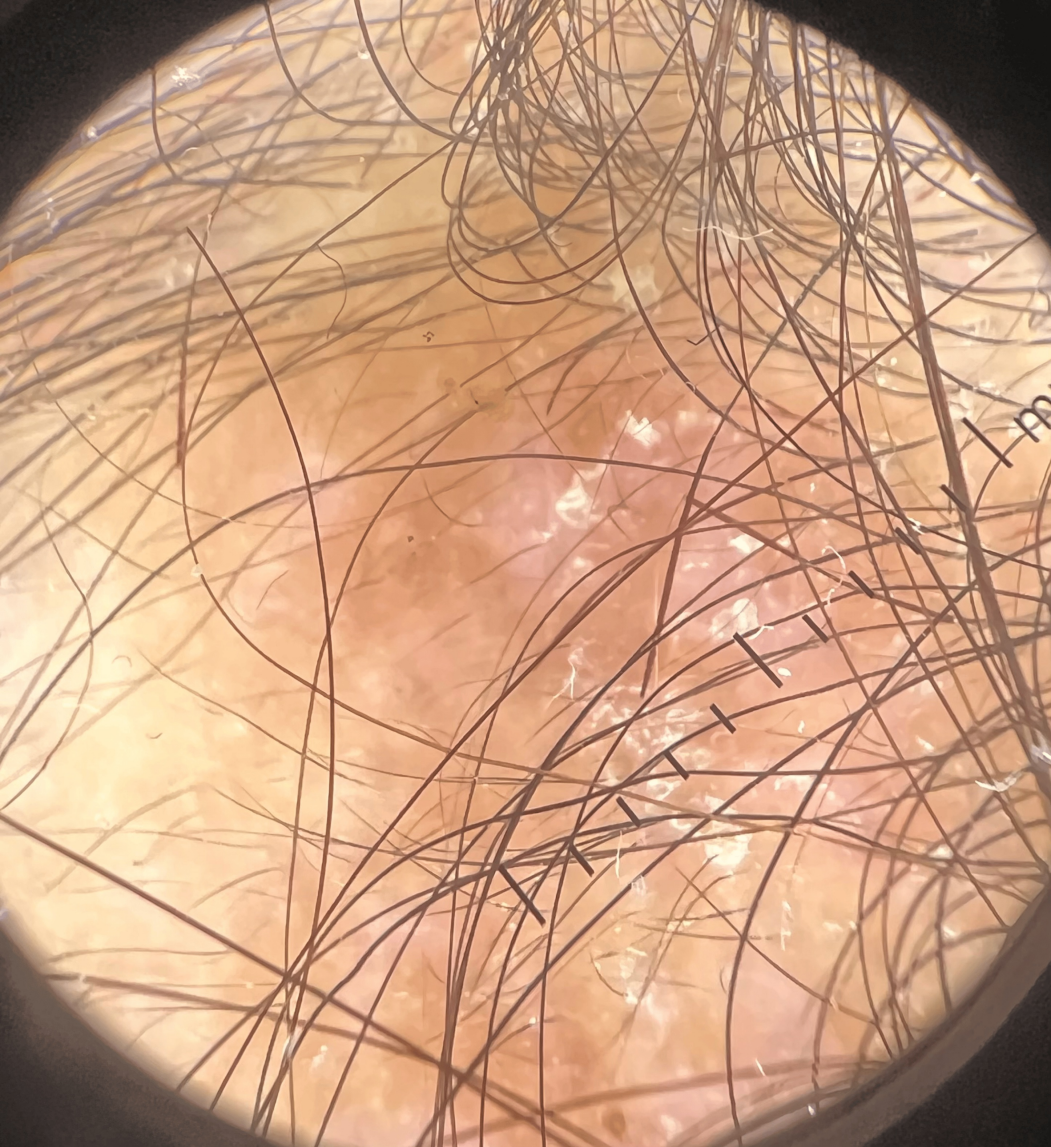
Dermoscopy of lichen planopilaris demonstrating perifollicular erythema, scaling, and peripilar casts, associated with reduced follicular openings and early fibrotic white areas.

**Figure 9:**
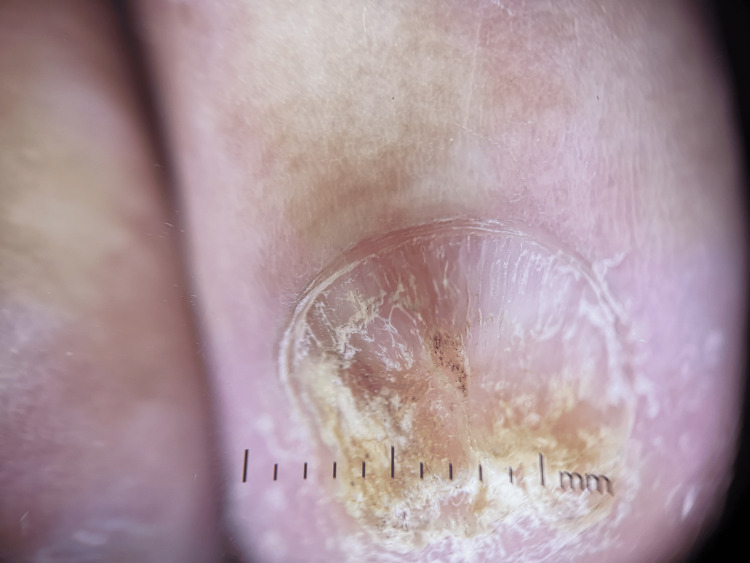
Dermoscopy of nail lichen planus demonstrating longitudinal ridging and fissuring associated with distal subungual hyperkeratosis and nail plate thinning.

These dermoscopic findings are not merely descriptive. They have practical value because they provide a visual bridge between morphology and pathophysiology. In pigmentary forms, the brown structures suggest melanophages and interface damage (Figure 6). In classic lichen planus, Wickham striae remain the best-known clue to diagnosis (Figure 7). In scalp disease, perifollicular inflammatory changes may help identify active lesions before irreversible scarring becomes prominent (Figure 8). Nail dermoscopy, although less standardized than skin dermoscopy, may also orient diagnosis and support early intervention (Figure 9).

Associated conditions were uncommon but noteworthy. Alopecia areata, vitiligo, and linear morphea were each observed in 2.77% of cases. These associations are clinically interesting because they support the notion of an autoimmune or dysimmune background in at least some patients.

Regarding treatment, several therapeutic approaches were used according to the clinical form and severity. What clearly emerges from the overall experience is that therapeutic response was only partially satisfactory and that most available therapies still lack robust evidence in pediatric disease. Associated diseases are summarized in Table 4.

**Table 4:** Associated diseases Data are presented as n (%).

Associated condition	Frequency
Alopecia areata	1 (2.77%)
Vitiligo	1 (2.77%)
Linear morphea	1 (2.77%)

## Discussion

Pediatric lichen is an uncommon inflammatory dermatosis, and our findings are consistent with previous reports highlighting the rarity of this condition in routine clinical practice [1,2]. This observation further highlights its low frequency in routine pediatric dermatology practice, even within a tertiary care center over an extended inclusion period. The inclusion of 36 pediatric cases over nearly 15 years reflects both the low incidence of this condition and its likely underrecognition, particularly in atypical or pauci-symptomatic forms. Most available studies report limited sample sizes, highlighting the absence of large prospective pediatric cohorts and the continued reliance on retrospective analyses, small case series, or systematic reviews [1,2,6].

The mean age at diagnosis in our study (9.9 ± 3.2 years) is consistent with previous reports describing a predominance of lichen in school-aged children [6,7]. The female predominance observed in our cohort may be explained by the higher frequency of certain subtypes, particularly lichen sclerosus, which has been reported more frequently in girls [7,8]. The relatively long duration before diagnosis observed in our study suggests that the disease may remain unrecognized for extended periods before specialized evaluation and reflects the diagnostic challenges associated with pediatric lichen. As previously reported, the polymorphic presentation of the disease, especially in pigmentary, nail, or follicular forms, may lead to confusion with other inflammatory or pigmentary dermatoses and contribute to delayed diagnosis [3,4,9].

One of the major findings of our study is the distribution of lichen subtypes observed in this cohort, including lichen sclerosus and pigmentary variants. This distribution of subtypes highlights the predominance of non-classical forms in this Moroccan pediatric cohort. This contrasts with several studies in which classical lichen planus is the most frequently reported subtype in children [6,10]. Such differences may be explained by geographic factors, variations in skin phototypes, referral bias in tertiary care settings, and differences in case definition across studies. In particular, studies conducted in populations with darker phototypes have reported a higher frequency of pigmentary forms, suggesting that melanin-related factors may influence disease expression and visibility [9,11].

These findings deserve particular attention. The predominance of lichen sclerosus suggests that pediatric lichen in this setting does not fully reflect the classical adult pattern, which is mainly centered on conventional lichen planus [12]. In addition, the relatively high proportion of lichen pigmentosus may be related to the phototype distribution of the cohort and the greater visibility of pigmentary disorders in children with intermediate to dark skin [9,11]. Furthermore, the frequency of nail involvement is clinically relevant, as nail disease in children is often overlooked or misdiagnosed despite its potential for permanent dystrophy [4].

Localization varied according to subtype, further underscoring the polymorphic nature of pediatric lichen and the importance of a systematic full-body examination in affected children. A comparison with published pediatric series further supports this heterogeneity. While some retrospective cohorts emphasize the predominance of classic lichen planus, others highlight the relative importance of lichen sclerosus or other clinicomorphologic variants depending on the study population [1,6,7,10]. This variability is important to consider because it directly affects both diagnostic reasoning and therapeutic strategy. In our setting, the predominance of non-classical forms suggests the need for clinicians to go beyond the traditional image of childhood lichen as merely “classic lichen planus” and to actively search for adnexal, pigmentary, and genital presentations [8,11,13].

A comparison with selected literature is summarized in Table 5.

**Table 5:** Comparative analysis of pediatric lichen in published studies

Study	Study design	Sample size	Predominant subtype	Key observations
Present study	Retrospective	36	Lichen sclerosus	Predominance of non-classical forms; frequent pigmentary and adnexal involvement
Merhy et al. [1]	Systematic review	985	Lichen planus	Wide clinical heterogeneity; low prevalence in children
Ravikiran et al. [6]	Retrospective	76	Lichen planus	Classical form predominant; mucocutaneous involvement common
Elloudi et al. [7]	Case series	10	Mixed forms	Rare condition; variable clinical presentation
Benassaia et al. [9]	Clinical study	-	Lichen planus	Interest of systemic retinoids in selected pediatric cases

Dermoscopy has emerged as a useful non-invasive diagnostic tool in inflammatory dermatoses, and its usefulness in lichen has been reported [5]. In our study, dermoscopy provided additional diagnostic clues and helped characterize the different clinical subtypes. In lichen pigmentosus, we observed a diffuse brown background with brown dots and globules, consistent with previously described dermoscopic features [11,14]. In classical lichen planus, Wickham striae were the most characteristic finding, often associated with vascular structures [13,15]. In lichen planopilaris, perifollicular erythema, scaling, and peripilar casts were observed, reflecting active inflammation and early scarring changes [14,16]. These findings are consistent with previous studies and support the potential integration of dermoscopy into routine clinical practice.

In children with skin of color, dermoscopic interpretation requires particular attention because pigmentation may modify the visibility and prominence of dermoscopic structures. Compared with lighter skin, darker phototypes more frequently show brownish backgrounds and darker structures, while vascular features may be less evident. In our cohort, phototypes IV (21 (58.82%)) and III (15 (41.17%)) accounted for all cases, which may partly explain the prominence of pigmentary findings such as diffuse brown background, brown dots, globules, and reticular pigmentation. These observations are consistent with recent data in pediatric skin of color, which highlight a predominance of darker phototypes and emphasize the increased visibility of pigmentary structures and relative paucity of vascular features compared with Caucasian populations [18]. In contrast, studies conducted in predominantly Caucasian populations more frequently describe vascular patterns and Wickham striae as prominent dermoscopic features [13,15]. These considerations highlight the importance of interpreting dermoscopic findings according to skin phototype rather than extrapolating directly from data derived mainly from lighter skin.

In pediatric patients, dermoscopy is particularly advantageous because it allows non-invasive evaluation of lesions and reduces the need for biopsy, which may be difficult to perform and poorly tolerated in children [5,16]. Furthermore, dermoscopy facilitates disease monitoring and assessment of therapeutic response, which may contribute to improved patient management. Its contribution is especially relevant in pigmentary and follicular forms, in which clinical morphology may be less specific, and histopathology may not always be immediately feasible [14,18].

The association of lichen with other dermatological conditions, such as alopecia areata, vitiligo, and morphea, although limited in our series, supports the hypothesis of an underlying autoimmune or dysimmune mechanism. Similar associations have been reported in the literature and suggest that lichen may be part of a broader spectrum of immune-mediated disorders [19,20]. From a pathophysiological perspective, lichen planus is characterized by a T-cell-mediated immune response directed against basal keratinocytes, resulting in interface dermatitis and apoptosis of keratinocytes [21]. This immunologic background may partly explain why overlap with other autoimmune skin diseases is occasionally observed in pediatric patients [20,21].

From a therapeutic standpoint, management remains challenging because of the lack of standardized guidelines in pediatric populations. Topical corticosteroids remain the first-line treatment, particularly for localized forms, but their efficacy may vary depending on disease severity, localization, and subtype [7,8]. In more extensive or refractory cases, systemic treatments such as retinoids may be considered. Systemic retinoids such as acitretin have been used in the management of lichen planus [12]. Other therapeutic options, including topical calcineurin inhibitors and immunomodulatory agents, have been proposed, but robust pediatric data remain scarce, and most recommendations are extrapolated from adult experience or small series [8,12].

Another important aspect is the potential long-term impact of the disease. Certain localizations, particularly scalp and nail involvement, may lead to irreversible sequelae if diagnosis and treatment are delayed [3,4]. Genital forms may also significantly impair quality of life because of chronic symptoms, discomfort, and the fear of visible or functional consequences. These elements reinforce the importance of early diagnosis, regular follow-up, and individualized management, particularly in children and adolescents [4,8,20].

Several limitations must be acknowledged. The retrospective design necessarily exposes the study to incomplete data capture and heterogeneity in clinical documentation. Because the data derive from routine practice over a long period, some variables were better detailed than others, and some therapeutic information could not be quantified precisely. The sample size, although respectable for a rare pediatric disorder, remains modest for robust subgroup comparisons. In addition, histologic confirmation was performed when necessary rather than uniformly in every case, which reflects real-life practice but may introduce some variability in diagnostic certainty across subtypes. The retrospective design may also limit the standardization of diagnostic criteria and interobserver reproducibility. The lack of standardized outcome measures may also limit the comparability of results. The retrospective design may also expose the study to information bias related to variability in clinical documentation. Finally, the tertiary-care setting may enrich the cohort for more atypical, persistent, or difficult cases and therefore may not perfectly reflect community prevalence or phenotype distribution.

Despite these limitations, the study has clear strengths. It spans nearly 15 years, includes a clinically meaningful number of children for a rare disease, documents several subtypes within the same institution, and incorporates dermoscopy in a structured way. It also contributes North African data, which remain underrepresented in the pediatric lichen literature. For these reasons, the manuscript adds value beyond a simple descriptive series and supports the need for larger multicenter pediatric studies with harmonized classification and outcome assessment.

## Conclusions

Pediatric lichen is a rare and heterogeneous inflammatory dermatosis with a wide spectrum of clinical presentations that extend beyond classical lichen planus. In our series, lichen sclerosus was the most frequent subtype, followed by pigmentary and nail forms, suggesting a predominance of non-classical phenotypes in children. The relatively long delay before diagnosis highlights the diagnostic challenges in pediatric patients, particularly in atypical, pigmentary, or adnexal presentations. These findings underline the importance of thorough clinical examination and increased awareness among clinicians to improve early recognition and potentially reduce the risk of complications, including permanent sequelae in nail and scalp involvement.

Dermoscopy appeared to be a useful non-invasive tool, providing additional diagnostic clues and facilitating the differentiation between various clinical subtypes. Its integration into routine clinical practice may improve diagnostic accuracy and could help reduce the need for invasive procedures in children. However, therapeutic management remains insufficiently standardized, reflecting the lack of robust pediatric data. Further multicenter studies are needed to better define diagnostic criteria and optimize treatment strategies in pediatric lichen.
